# Comment on Pop et al. Early Diagnosis of Oral Mucosal Alterations in Smokers and E-Cigarette Users Based on Micronuclei Count: A Cross-Sectional Study among Dental Students. *Int. J. Environ. Res. Public Health* 2021, *18*, 13246

**DOI:** 10.3390/ijerph19063713

**Published:** 2022-03-21

**Authors:** Wilton Mitsunari Takeshita, Daniel Araki Ribeiro

**Affiliations:** 1 Department of Dentistry, Federal University of Sergipe, UFS, Aracaju 49060-108, SE, Brazil; wmtakeshita2@gmail.com; 2 Department of Biosciences, Institute of Health and Society, Federal University of São Paulo, UNIFESP, Santos 11015-020, SP, Brazil

We read the manuscript recently published in *International Journal of Environmental Research and Public Health* titled “Early diagnosis of oral mucosal alterations in smokers and e-cigarette users based on micronuclei count: a cross-sectional study among dental students” by Pop et al. [1] with much interest. In the study, the authors proposed the application of a micronucleus assay as a suitable tool for detecting cytogenetic damage in buccal cells. In the study, the authors found a high number of micronuclei in smokers and e-cigarette users when compared to non-smokers (control group). However, some theoretical concepts and guidelines are described for the correct understanding of the manuscript.

In the manuscript, it was written that all specimens were stained with Papanicolaou stain (Pap stain). In the Discussion, it was stated that “Therefore, based on the acceptable accuracy regarding MN evaluation, Pap stain may be used for routine screening and samples with potential abnormalities can be further processed with DNA-specific stains”. It is important to highlight that Pap stain is not the best possible option for micronucleus testing because there are structures in the cytoplasm of buccal cells that are identical to micronuclei, such as, for example, cytoplasmic granules or inflammatory cells, and it is therefore, impossible to identify it with accuracy [2]. Moreover, a figure in the range from 0.3 to 1.7/1000 was considered to be correct in considering the spontaneous micronucleus incidence in buccal cells by the micronucleus assay guidelines [2]. The data presented in the manuscript for the control group in MC count and MNC count were 1.95 ± 1.05 and 1.4 ± 0.68, respectively. Certainly, these results were related to false-positive data.

As usual, all Tables and Figures should be self-explanatory, i.e., they should provide clear presentations and syntheses of the results found. Herein, what does Groups A–C, MN and MNC presented in Tables 1–3 and Figure 3 mean? This information is present only in the Results. In Table 2 and Figure 2, the authors indicated the analysis of micronucleus frequency by means of the total number of micronuclei (MN) and total micronucleated cells (MNC). What is the biological and clinical relevance of the approach? Taking into consideration the micronucleus assay guidelines, there is not any significance linked to the total number of micronucleus in the any eukaryotic cell.

In Material and Methods, a total of 1000 buccal cells were counted per volunteer. Following the guidelines proposed by the International Micronucleus Assay Group, the analysis of 2000 cells per person has been established [2]. Certainly, the approach would dramatically improve the quality of the study data.

Interestingly, the Discussion starts with the phrase “The aim of this study was to determine whether smokers or e-cigarette users exhibit cytotoxic damage of the buccal mucosa in the absence of any significant clinical manifestation noticeable to the individual, which could be a false indicator of wellbeing”. In our opinion, the statement does not make any sense, since the presence of a micronucleus suggests mutagenic effects as a result of chromosome breakage, and not cytotoxicity.

Finally, it is worth mentioning that Tolbert et al. [3] have revealed the presence of metanuclear changes, suggesting the presence of cytotoxicity in buccal cells, such as for example, karyorrhexis, pyknosis and karyolysis. The approach is valid and necessary because cytotoxicity interferes with the amount of micronuclei. If cytotoxicity is positive, the micronucleated cells are subtracted at the expense of cellular death. Particularly, this is clearly seen in the oral cells from chronic smokers [4,5,6].

We assumed that such comments are useful for the correct understanding of the manuscript validating the micronucleus assay in smokers and e-cigarette users as a suitable tool using buccal cells. 

## Data Availability

Not applicable.
